# Mesenchymal Stromal Cells Affect Disease Outcomes via Macrophage Polarization

**DOI:** 10.1155/2015/989473

**Published:** 2015-07-15

**Authors:** Guoping Zheng, Menghua Ge, Guanguan Qiu, Qiang Shu, Jianguo Xu

**Affiliations:** ^1^Shaoxing Second Hospital, Shaoxing, Zhejiang 312000, China; ^2^The Children's Hospital of Zhejiang University School of Medicine, Hangzhou, Zhejiang 310052, China; ^3^The First Affiliated Hospital of Zhejiang University School of Medicine, Hangzhou, Zhejiang 310003, China

## Abstract

Mesenchymal stromal cells (MSCs) are multipotent and self-renewable cells that reside in almost all postnatal tissues. In recent years, many studies have reported the effect of MSCs on the innate and adaptive immune systems. MSCs regulate the proliferation, activation, and effector function of T lymphocytes, professional antigen presenting cells (dendritic cells, macrophages, and B lymphocytes), and NK cells via direct cell-to-cell contact or production of soluble factors including indoleamine 2,3-dioxygenase, prostaglandin E2, tumor necrosis factor-*α* stimulated gene/protein 6, nitric oxide, and IL-10. MSCs are also able to reprogram macrophages from a proinflammatory M1 phenotype toward an anti-inflammatory M2 phenotype capable of regulating immune response. Because of their capacity for differentiation and immunomodulation, MSCs have been used in many preclinical and clinical studies as possible new therapeutic agents for the treatment of autoimmune, degenerative, and inflammatory diseases. In this review, we discuss the central role of MSCs in macrophage polarization and outcomes of diseases such as wound healing, brain/spinal cord injuries, and diseases of heart, lung, and kidney in animal models.

## 1. Introduction

MSCs are nonhematopoietic cells with multipotent capacity in the bone marrow (BM) and in the connective tissues of most organs. The revolutionary findings for MSCs came from Friedenstein et al. in 1970, when they first reported the development of fibroblast colonies in monolayer cultures of guinea-pig BM and spleen cells [1]. The term “mesenchymal stem cells,” a synonym for MSCs, was first coined in 1991 by Caplan [2]. MSCs did not receive wide attention until Pittenger et al. demonstrated their multilineage potential [3]. In addition to their differentiation properties, MSCs possess broad immunoregulatory properties [4]. Their capacities for differentiation and immunoregulatory functions have made them an outstanding candidate in the clinical treatment of many pathologic conditions in which inflammation and immunopathologic reactions have a fundamental role [5]. These diseases include graft-versus-host diseases (GVHD) [6], autoimmune diseases such as Crohn's disease [7], myocardial infarction (MI) [8], osteoarticular diseases [9], and acute respiratory distress syndrome published from our group [10].

Apart from MSCs, other types of stem cells such as embryonic stem cells (ESCs) and induced pluripotent stem cells (iPSCs) have also raised the hope for future regenerative medicine. Human ESCs (hESCs) have the ability to differentiate into all types of adult human tissues and to grow indefinitely [11]. However, isolating hESCs involves the destruction of the blastocyst, which is a controversial ethical issue. The safety and effectiveness of ESCs have been tested in clinical trials for age-related macular degeneration and Stargardt's macular dystrophy [12]. The application of iPSCs has been tested in mouse models of Parkinson's disease [13] and sickle cell anemia [14]. However, clinical application of iPSCs is another controversial topic due to the potential for iPSCs to form tumors via oncogene activation and insertional mutagenesis [15]. The first clinical trial using iPSCs is currently being conducted in Japan for age-related macular degeneration.

It has been reported that MSCs can exert their inhibitory effect on both adaptive and innate immunity via both cell contact-dependent mechanisms and soluble factors [16]. For adaptive immunity, MSCs have been reported to decrease the secretion of inflammatory cytokines by various immune cell populations [17]. MSCs strongly inhibit T-cell proliferation both* in vitro* and* in vivo* [18] and induce T-cell division arrest [19]. In addition, it has been shown that MSCs suppress not only Th1 functions but also the Th17-mediated activation and proliferation through soluble and cell-dependent factors [20]. Furthermore, MSCs modulate B-cell functions by suppressing B-cell terminal differentiation and plasma cell immunoglobulin production [21, 22]. For innate immunity, MSCs have been documented to inhibit NK cell proliferation, cytotoxicity, and cytokine production via indoleamine 2,3-dioxygenase (IDO) and prostaglandin E2 (PGE2) [23]. MSCs decrease the generation and function of dendritic cells, an essential of antigen-presenting cells, and induce T-cell unresponsiveness [24]. Many studies have demonstrated that MSCs would interfere with the acquisition of M1 macrophage phenotype, while promoting M2 polarization [25]. In this review, we highlight the role of MSCs in macrophage polarization in multiple disease models, which has not been reviewed comprehensively.

## 2. MSCs: General Properties

MSCs are adult, fibroblast-like multipotent cells characterized by the ability to differentiate [26]. The wide variety of origins, preparation methodologies, and nomenclature prompted standardization in 2006 by the International Society for Cellular Therapy, which set three minimum requirements for MSCs definition [27]. First, MSCs have plastic adherent capacity. Second, MSCs must bear certain stromal surface markers (CD73, CD90, and CD105) and lack hematopoietic cell markers such as CD11b, CD14, CD19, CD34, CD45, and major histocompatibility complex (MHC) class II. In addition, they should have adipogenic, osteogenic, and chondrogenic differentiation potential [27]. In addition to the minimum requirements, MSCs also have the ability to differentiate into cells not only in the mesenchymal lineage but also cells in ectodermal (epithelial cells and neuroglial-like cells) and endodermal (lung cells, gut epithelial cells, and hepatocyte-like cells) lineages [3, 28]. However, transdifferentiation is a rare event* in vivo*. For example, it has been reported that transdifferentiation of MSCs to pancreatic *β*-cells is very limited or nonexistent in animal model of diabetes treated with MSCs [29].

The BM has been historically the prime source of MSCs. However, MSCs comprise only a minor fraction of BM tissues, with BM-MSCs constituting a mere 0.0001%–0.01% of all BM-nucleated cells [3]. Because of their ease of procurement and the abundance, MSCs from the adipose tissue and umbilical cord blood have garnered significant attention over the past few years [30, 31]. The differences concerning the morphology and immune phenotype of the MSCs derived from these three sources are not significant. However, there were reported variations in colony frequency and proliferation capacity for MSCs among the three sources [32].

Recently, Waterman et al. proposed that MSCs are not constitutively immunosuppressive and can be classified into two phenotypes including proinflammatory MSC1 and immunosuppressive MSC2 [33]. They demonstrated that MSCs adopted a proinflammatory phenotype (MSC1) with elevated levels of proinflammatory cytokines such as IL-6 and IL-8, when human MSCs were primed with LPS, the ligand for Toll-like receptor 4 (TLR4). On the other hand, TLR3-primed human MSCs adopted an immunosuppressive phenotype (MSC2) and expressed mostly immunosuppressive cytokines such as CCL10 (IP10), CCL5 (RANTES), and IL4. MSC2, but not MSC1, had an increased expression of known immune suppressive effectors such as IDO and PGE2. Furthermore, MSC1 supported T-cell activation, while unprimed MSCs and MSC2 suppressed it [33]. However, their findings have not been confirmed by others. Another feature of MSCs is that they express low levels of cell-surface human leukocyte antigen (HLA) class I molecules. In addition to being MHC II negative, MSCs do not appear to express the costimulatory molecules CD40, CD40L, CD80, or CD86 required for effector T-cell induction. Therefore, MSCs are considered to be immune-privileged [34–36]. These findings support that MSCs can be transplantable between HLA-incompatible individuals. Therefore, therapeutic potency, safety, and efficacy of the treatment with MSCs might reside to a large extent in their immunologically privileged phenotype and in their immunomodulatory capacity.

## 3. MSCs: Mechanisms of Action

MSCs act via multifaceted pathways that are not fully understood. Since MSCs have the ability to differentiate into various cell types, it was initially thought that engraftment and differentiation into injured tissues were the mechanisms for their regenerative properties. In fact, engrafted MSCs have been identified at sites of injury along with improvements in regeneration and function [37, 38]. However, engraftment is a rare event and it is difficult to correlate the extent of limited engraftment with dramatic functional improvement. Currently, the main proposed mechanisms through which MSCs display their reparative/regenerative effects after tissue damage include the capacity to home to sites of injury, the ability to release anti-inflammatory soluble factors, and the immunomodulatory property [39]. These mechanisms are described in the following paragraphs in detail except for MSCs-mediated regulation on macrophages, which is discussed in another section.

For homing of MSCs, intravenous infusion of MSCs often results in the entrapment of the administered cells in the organ capillary beds of the lung [40]. Following injury, MSCs have the capacity to migrate along an inflammatory cytokine gradient and home to the site of damage, governed largely by chemokines and their receptors [41]. For example, stromal cell-derived factor-1 (SDF-1)/CXCR4 pathway has been shown to mediate the localization of injected MSCs into the injured kidneys [42]. Although tissue-specific homing has been reported in many conditions, long-term engraftment of the MSCs is not a common event. Therefore, many investigators are trying to enhance the MSC's migratory properties, survival, and regenerative capacity through preconditioning with growth factors, exposure to hypoxia, or genetic modification [43–45]. Second, MSCs have been documented to decrease the proinflammatory cytokines (IL-1*β*, TNF-*α*, IFN-*γ*, IL-6) and increase anti-inflammatory cytokines (IL-10, basic fibroblast growth factor, TGF-*α*, TGF-*β*) in alleviating acute injury of the kidney [46], liver [47], and lung [48]. Similar findings were also reported in animal models of stroke [49] and sepsis [50]. Third, MSCs can modulate innate and adaptive immune cells, by enhancing anti-inflammatory pathways in the injured organ milieu [51]. This immunomodulation is mediated through the release of soluble factors such as IDO [52], PGE2 [53], TGF-*β* [54], IL-10 [53], IL-1 receptor antagonist [55], HGF [18], TNF-*α* stimulated gene 6 (TSG-6) [56], and NO [57] and/or through cell contact signaling such as Notch and CD95/Fas [58, 59].

One of the major mechanisms of immunomodulation by MSCs is the regulation on T cells. First, MSCs are able to suppress T-cell proliferation induced by cellular or nonspecific mitogenic stimuli [18] and inhibit the response of naive and memory antigen-specific T cells [60]. The inhibitory effect is independent of antigen presenting cells, MHC molecules, and regulatory T cells (Treg) [35]. MSCs suppress T-cell proliferation primarily via the secretion of soluble factors such as TGF-*β*1 [18], HGF [18], IL-10 [24], and PGE2 [17]. In addition, IDO [61] and NO [57] are also involved in the process [57]. For example, the release of IFN-*γ* by damaged target cells is able to induce the release of IDO by MSCs, which, through the depletion of tryptophan, results in an antiproliferative effect [62]. Secondary, MSCs are also able to decrease T-cell response by shifting them from a T helper (Th)1 to a Th2 immune phenotype [18]. Modification of Th1/Th2 balance by MSCs has been demonstrated in a model of ragweed-induced allergic asthma. MSCs administration was associated with reduced levels of IL-4 and IL-13 in serum and bronchioalveolar lavage fluid as well as deceased levels of IgG_1_, IgE, inflammatory cell infiltration, and mucus deposition in the lungs. IL-4 and/or IL-13 activated the STAT6 pathway in the MSCs resulting in an increase of TGF-*β*1 production, which might mediate the beneficial effect, either alone or together with Treg recruited by the MSCs [54]. Thirdly, MSCs are able to inhibit Th17 cell differentiation and function and induce a Treg phenotype [20]. The inhibition of Th17 cell differentiation is triggered by cell-to-cell contact and mediated by PGE2 via the EP4 receptor [63]. A number of studies have shown the capacity of MSCs to promote the generation of Treg by activating the Notch 1 signaling pathway [64] or through production of HLA-G5 [65]. Also, MSCs-derived PGE2 and TGF-*β*1 were shown to have a nonredundant role in the induction of CD4+CD25+FoxP3+ Treg. Furthermore, purified Treg induced by MSCs coculture were able to suppress alloantigen-driven proliferative responses in mixed lymphocyte reaction [66]. In a renal transplantation model, MSCs were shown to induce the generation of Tregs and kidney allograft tolerance via secretion of IDO [52]. The involvement of Treg in mediating beneficial effects of MSCs in several disease states was confirmed via Treg depletion [52, 67]. Finally, MSCs were shown to suppress CD8+ cytotoxic T-cell activation via secretion of PGE2, IDO, and TGF-*β* [68].

MSCs can inhibit generation and function of both CD34 derived and monocyte-derived dendritic cells (DC) [69]. DC cocultured with MSCs were arrested in the G0/G1 phase as a result of cyclin D2 downregulation [70]. Mature DC treated with MSCs became immature and had reduced expression of CD83. Meanwhile, the expression of presentation molecules (HLA-DR and CD1a), costimulatory molecules (CD80 and CD86), and IL-12 secretion was also decreased. Functionally, the allostimulatory ability of DC on allogeneic T cells was impaired [71]. MSCs also transform DC into anti-inflammatory or tolerogenic phenotypes, decrease TNF-*α* secretion in DC1, and increase IL-10 secretion in DC2 [17]. Mechanistically, it has been reported that MSCs inhibit DC maturation via IL-6 [69]. Other studies have identified a role for the Notch signaling pathway in DC differentiation [59].

MSCs also modulate NK cells by altering the phenotype of NK cells and suppressing proliferation, cytokine secretion, and cytotoxicity against targets [72]. This inhibitory effect of MSCs was associated with downregulated expression of the activating NK cell receptors and mediated by PGE-2, IDO, and TGF-*β*1 [23]. Initially, it was thought that MSCs could escape NK cell immunosurveillance [73]. Subsequent reports showed that activated NK cells can also efficiently lyse MSCs by engaging their activating receptors: NKp30, NKG2D, and DNAM-1 [74]. Recently, MSCs were shown to adapt their immunobehavior in an inflammatory context, decreasing their susceptibility to NK killing. In addition, TLR3 but not TLR4-primed MSCs enhanced their suppressive functions against NK cells [75].

## 4. M1 and M2 Macrophages

Macrophages are an essential component in the orchestration and expression of innate immunity and adaptive immune responses. These cells play a central role in inflammation and host defense. Additionally, cells of the monocyte-macrophage lineage fulfill homeostatic functions beyond defense [76]. These functions include tissue repair, wound healing, and regulation of metabolic activity [77]. The function of macrophages is tailored to their tissue of residence, an adaptation that is driven by tissue-derived factors and by the physiological environment [76]. Depending on the microenvironment, macrophages can acquire distinct functional phenotypes. The concept of macrophage polarization was first described in 1992 with the discovery that IL-4 potently enhances murine macrophage mannose receptor (CD206) activity [78]. Since then, two opposite and competing phenotypes, often referred to as classically activated macrophages (M1 macrophages) and alternatively activated macrophages (M2 macrophages), have been defined and identified in several physiological settings [79]. Although the classification of macrophages as M1/M2 came after the classification of lymphocytes into Th1 and Th2, the Th1 and Th2-like responses are the results of polarization of macrophages to M1 and M2 states, respectively. Furthermore, M1/M2 polarization is not dependent on T cells, as has been demonstrated in Rag-1 knockout and other immune deficient mice [80].

M1 macrophages are induced by TLR ligands (such as lipopolysaccharide, LPS) and IFN-*γ*. They express higher levels of CD86 and PD-L1 [81]. M1 macrophages are characterized by increased microbicidal activity and produce several proinflammatory mediators, such as inducible nitric oxide synthase, TNF-*α*, IL-1*β*, IL-6, IL-12, and proteolytic enzymes [82]. They constitute the first line of defense against pathogens and promote Th1 polarization of CD4+ lymphocytes. On the other hand, M2 macrophages are induced by Th2-type cytokines, such as IL-4 and IL-13. Tregs have also been implicated in the induction of M2 polarization, possibly through IL-10 [83]. M2-like cells have been described in different pathological conditions such as infections by intracellular bacteria or virus, allergy, diabetes, and cancer [79, 84]. They are characterized by the expression of CD163, CD206, arginase 1, FIZZ1 (found in inflammatory zone 1), and CD36. In addition, they secrete anti-inflammatory cytokines, such as TGF-*β*, IL-1 receptor antagonist, and IL-10. M2 macrophages play an essential role in the suppression of Th1 immune responses and the enhancement of tissue remodeling and Th2 response [85]. Macrophages with intermediate or overlapping phenotypes have also been reported. For example, adipose tissue macrophages from obese mice have a mixed profile, with upregulation of several M1 and M2 gene transcripts [86].

## 5. Mechanisms of MSCs-Mediated Regulation on Monocytes/Macrophages

Kim and Hematti were the first to report that MSCs could polarize macrophages from the classic proinflammatory M1 phenotype, toward the anti-inflammatory M2 phenotype [25]. They found that macrophages cocultured with MSCs consistently showed a high level expression of markers for M2 macrophages. The resulting macrophages produced high levels of IL-10 and low levels of IL-12 and TNF-*α*. Functionally, macrophages cocultured with MSCs showed a higher level of phagocytic activity. Other studies showed that MSC-mediated polarization of M2 macrophages depends on the secretion of soluble factors, including PGE2, TSG-6, IL-6, IDO, and TGF-*β*1 [53, 87–89].

Using a model of cecal ligation and puncture, Németh et al. demonstrated that BM-MSCs attenuate sepsis via cyclooxygenase-2 and PGE2–dependent reprogramming of host macrophages to increase their IL-10 production [53]. They propose that MSCs are activated by LPS or TNF-*α*. Then, MSCs reprogram macrophages by increasing cyclooxygenase-2 activity and releasing PGE2 that acts on the macrophages through the prostaglandin receptors. Next, activated macrophages produce anti-inflammatory IL-10 which reduces inflammation. In an experimental model of zymosan-induced peritonitis, MSCs were reported to be activated by inflammatory signals to secrete the anti-inflammatory protein, TSG-6 [87]. TSG-6 interacted through the CD44 receptor on resident macrophages to decrease zymosan/TLR2 signaling. The negative feedback loop created by MSCs through TSG-6 attenuated the inflammatory response that was initiated by resident macrophages. These results may explain the beneficial effects of MSCs and TSG-6 in several disease models in which inflammation is the underling mechanism.

**Figure 1:**
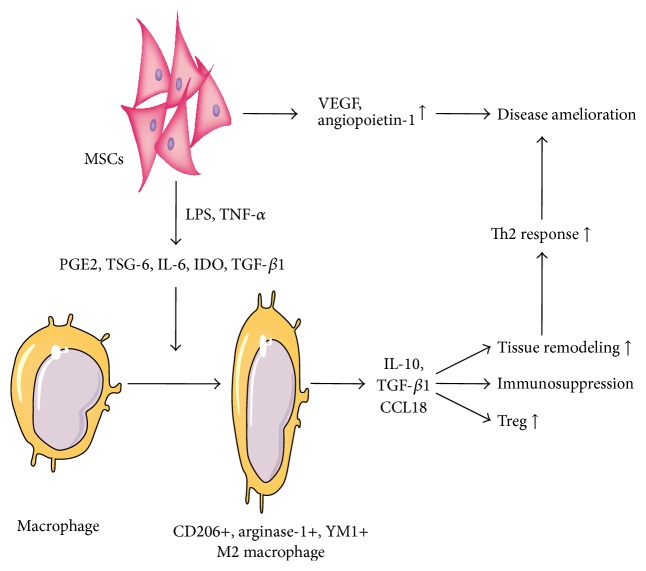
Mechanisms of macrophage polarization induced by MSCs in disease amelioration. MSCs produce paracrine factors such as PGE2, TSG-6, IL-6, IDO, and TGF-*β*1 when stimulated by proinflammatory mediators (LPS and TNF-*α*). The paracrine factors promote M2 macrophage polarization, which leads to increased Th2 response (upregulation of Treg and tissue remodeling as well as immunosuppression). MSCs also facilitate the production of VEGF and angiopoietin-1 which enhance angiogenesis. The net effect is disease amelioration.

Melief et al. reported that the addition of MSCs to monocyte cultures prevented the differentiation of monocytes towards antigen-presenting immunogenic cells and skewed differentiation towards an anti-inflammatory IL-10-producing cell type [88]. The coculture supernatants contained higher concentrations of IL-6 and IL-10. Neutralizing IL-6 antibody reversed the inhibitory effect of MSCs. Results from the Galipeau group demonstrated that MSCs from normal volunteers upregulated IDO expression in the presence of TNF-*α* and IFN-*γ* [89]. Elevated IDO activity was implicated in the differentiation of monocytes into IL-10-secreting M2 macrophages. Those monocyte-derived M2 macrophages were in turn implicated in the suppression of T-cell proliferation in an IL-10-independent manner, thus amplifying the immunosuppressive effect generated by MSCs. By depleting monocytes from peripheral blood, another* in vitro* study discovered that monocytes were essential for MSC-induced Treg formation [90]. They propose that MSCs promoted the survival of monocytes and induced differentiation toward M2 macrophages through unknown soluble factors. Then, M2 macrophages secreted high levels of IL-10 and CCL-18, which mediated the observed Treg induction (Figure 1).

## 6. MSCs-Induced M2 Macrophages in Wound Healing

Zhang et al. first described the interplay between MSCs and macrophages and the potential relevance in a splinted murine wound model [91]. When cocultured with gingiva-derived MSCs, macrophages acquired an anti-inflammatory M2 phenotype characterized by (1) an increased expression of CD206 and IL-10, (2) a decreased production of TNF-*α*, and (3) a reduced ability to induce Th-17 cell expansion.* In vivo*, MSCs homed to the wound site in close proximity with host macrophages, polarized toward the M2 phenotype, and enhanced wound repair. Mechanistically, MSCs treatment increased the expression of IL-10 and mitigated local inflammation by a decreased infiltration of inflammatory cells and a reduction of IL-6 and TNF-*α* levels. As a result, a significant enhancement of cutaneous wound healing was observed consisting of increased reepithelialization, collagen deposition, and angiogenesis. In another study using the same wound healing model, both MSCs from human umbilical cord and the conditioned medium accelerated wound healing by enhancing collagen deposition and angiogenesis [92]. Conditioned medium of the MSCs increased the number of anti-inflammatory M2 macrophages expressing resistin-like molecule- (RELM-) *α*/CD11b and promoted neovessel maturation. Conditioned medium also increased the expression of tissue-repairing cytokines including IL-10, TGF-*β*1, vascular endothelial growth factor- (VEGF-) 1, and angiopoietin-1. Adipose tissue-derived MSCs were shown to have the same effect on M2 macrophage polarization during wound healing [93]. When adipose tissue-derived MSCs were delivered to murine wounds by silicon carriers, wound healing was significantly accelerated, similar to delivery via intradermal injection. More than 80% of the MSCs were transferred from carriers to wounds in 3 days. Carrier-delivered MSCs were shown to increase anti-inflammatory M2 macrophages, TGF-*β*1-dependent angiogenesis, myofibroblast differentiation, and granulation tissue formation.

## 7. MSCs-Induced M2 Macrophages in Lung Diseases

Studies from our group [48] and others [94] have documented the benefits of MSCs in animal models of lung injury. M2 macrophage activation has been reported as one of the mechanisms of BM-MSCs in alleviating lung injury by Ionescu et al. [95]. They documented that both MSCs and MSCs-derived conditioned medium (MSCs-CdM) promoted the resolution of LPS-induced lung injury. MSCs-CdM increased arginase-1 activity and Ym1 expression in LPS-treated alveolar macrophages.* In vivo*, alveolar macrophages from lungs treated with LPS-MSCs and LPS-MSCs-CdM had elevated expression of Ym1 and decreased level of inducible nitric oxide synthase compared with that of untreated LPS mice. In the same model of LPS-induced lung injury, MSCs improved lung function through modulation of the inflammatory and remodeling processes [96]. There was a reduction in collagen fiber content associated with increased expression of metalloproteinase-8 and decreased metalloproteinase-1 expression in mice treated with MSCs. There was also a trend toward increased levels of Th2 mediators, IL-13 and IL-2, in lung tissue homogenate. Furthermore, arginase-1 expression in lung tissue was significantly enhanced after MSCs treatment. In a mouse model of experimental emphysema, intravenous administration of both BM- and adipose-derived MSCs decreased the number of M1 macrophages and pulmonary hypertension, while increasing VEGF levels. However, intravenous administration of BM-MSCs resulted in better cardiovascular function and a significant change in the macrophage phenotype from M1 to M2 [97].

## 8. MSCs-Induced M2 Macrophages in Heart Diseases

Dayan et al. studied the effect of MSCs in a mouse model of acute MI [98]. After infusion of MSCs, the overall macrophage/monocyte levels were reduced including both M1 and M2 macrophages. However, the proportion of M2 macrophages was significantly increased in the circulation and heart but not the BM. This was accompanied by a decreased expression of IL-1*β* and IL-6, increased IL-10 expression, and fewer apoptotic cardiomyocytes in the infarct area. Fractional shortening was elevated 2 weeks after cell infusion but was similar to medium controls 16 weeks after MI.* In vitro*, MSCs-mediated secretion of IL-10 was involved in the increased frequency of alternatively activated monocytes/macrophages. In a rat model of MI after coronary ligation, transplantation of MSCs sheets had significantly improved cardiac function and reduced myocardial fibrosis compared with the untreated MI group [99]. In MSCs sheet-transplanted groups, the peri-infarct regional capillary density was increased. Cell engraftment and differentiation were very low after MSCs sheet transplantation. CD163+ M2 macrophages were significantly increased at the transplantation site with no change in CD3+ T cells. In another MI study with mice, transient macrophage depletion increased mortality 30 days after MI with and without MSCs therapy [100]. Early macrophage depletion negatively affected infarct size, left ventricular function, and left ventricular thrombus formation. Furthermore, these adverse effects were ameliorated with macrophage restoration and MSCs therapy. The MSCs therapy significantly elevated the percentage of F4/80+CD206+ M2 macrophages in the infarcted myocardium as compared with control hearts, 3 and 4 days after MI. Th2 related cytokines, such as IL-10, IL-5, and GM-CSF, were significantly enhanced after MSCs therapy, or incubation with MSCs or MSCs supernatant. In addition to the studies with traditional sources of MSCs, human cardiac adipose tissue-derived MSCs (AT-MSCs) also favored the generation of M2 macrophages [101]. When cultured with human AT-MSCs, macrophages acquired the shape of M2 phenotype and increased the expression of M2 markers CD206+, CD163+, and CD16+. AT-MSCs increased the levels of anti-inflammatory IL-10 and angiogenic VEGF. AT-MSCs also decreased the levels of inflammatory cytokines such as IL-1*α*, TNF-*α*, IL-17, and IFN-*γ*.

## 9. MSCs-Induced M2 Macrophages in Renal Diseases

The involvement of macrophages in the therapeutic effect of human umbilical cord-derived MSCs has been reported in a model of renal ischemia-reperfusion injury [102]. Intravenous administration of MSCs resulted in a lower tubular injury score along with more proliferative and fewer apoptotic tubular cells at 24 h after reperfusion. The alleviated injury was accompanied by significant improvements in renal function. MSCs also decreased the infiltration of macrophages into renal interstitium at 5 days after reperfusion. However, the proportion of anti-inflammatory M2 macrophages was significantly elevated.* In vitro*, macrophages cocultured with human umbilical cord-derived MSCs acquired the alternatively activated M2 phenotype. Duffy et al. studied whether the immunomodulatory effect of MSCs on Th17 cells was enhanced by the addition of other agents in a mouse model of sterile kidney inflammation (unilateral ureteral obstruction) [103]. Coadministration of MSCs and paricalcitol, a vitamin D receptor (VDR) agonist, resulted in an early (day 3) reduction of intrarenal CD4(+) and CD8(+) T cells and neutrophils. It also decreased inflammatory monocytes as well as IL-17A levels as compared with untreated animals. At the late stage (day 8), mice double-treated with MSCs/paricalcitol had lower numbers of neutrophils and inflammatory monocytes and an increase in the ratio of M2/M1 macrophages as compared with control mice. Double treatment also decreased tubular injury and interstitial fibrosis. Another report by Wise et al. showed that MSCs altered macrophage phenotype and promoted regeneration via homing to the kidney following ischemia-reperfusion injury [104]. After intravenous administration of MSCs to mice with ischemia-reperfusion injury, MSCs homed to injured kidneys and decreased proximal tubule kidney injury molecule-1 expression, blood urea nitrogen, and serum creatinine levels. MSCs decreased collagen and elevated matrix metalloproteinase-9 activity in affected kidneys. Following direct and indirect coculture, macrophages elevated gene expression of an anti-inflammatory M2 phenotype including Arg1, Chi3l3, Ccl2, and Fizz1. Geng et al. demonstrated that a single intravenous infusion of MSCs given 6 hours after induction of acute muscle necrosis (rhabdomyolysis) robustly ameliorated renal function impairment and severe tubular injury [105]. Depletion of macrophages with clodronate delayed restoration of renal injury. There was also a time-dependent increase in intrarenal accumulation of CD206+ M2 macrophages. The beneficial effect of MSCs could be replicated by MSCs-educated macrophages.* In vitro*, macrophages cocultured with MSCs acquired an anti-inflammatory M2 phenotype with an increased expression of CD206 and IL-10.

## 10. MSCs-Induced M2 Macrophages in Spinal Cord and Brain Injury

Nakajima et al. reported that transplantation of MSCs promoted an alternative pathway of macrophage activation and functional recovery after spinal cord injury [106]. T9-T10 spinal cord injury was induced by contusion in rats. PKH26-labeled MSCs were transplanted into the contusion epicenter 3 days later. The transplanted MSCs migrated within the injured spinal cord without differentiating into glial or neuronal elements. MSCs transplantation was associated with significant increases in IL-4 and IL-13 levels and decreases in TNF-*α* and IL-6 levels. The MSCs group had increased numbers of M2 macrophages and decreased numbers of M1 macrophages in the injured region. These changes were accompanied by preserved axons, less scar tissue formation, and increased myelin sparing. These results indicate that MSCs transplantation after spinal cord injury modified the inflammatory environment by directing macrophages towards M2 phenotype and favored axonal extension and functional recovery. In another study of spinal cord injury, alginate microencapsulated MSCs were used for the study [107]. Rats were injured at the T10 vertebra via a weight drop model (NYU model) and encapsulated human MSCs were administered via lumbar puncture at 24 h after injury. Injected MSCs were mainly discovered in the cauda equina of the spinal cord. As few as 5 × 10^4^ encapsulated MSCs led to increased numbers of CD206-expressing macrophages in the spinal cord tissue 7 days after injury. In another report of rat spinal cord injury, intrathecal administration of conditioned-medium from MSCs of human deciduous dental pulp resulted in remarkable functional recovery during the acute phase [108]. Secretome analysis of the conditioned medium demonstrated a novel set of inducers for anti-inflammatory M2-like macrophages: monocyte chemoattractant protein-1 (MCP-1) and the secreted ectodomain of sialic acid-binding Ig-like lectin-9 (ED-Siglec-9). Depletion of MCP-1 and ED-Siglec-9 from conditioned medium decreased the ability to induce M2 macrophages and recover from spinal cord injury. Furthermore, macrophages treated with MCP-1 and ED-Siglec-9 extended neurites and inhibited the apoptosis of primary neurons. MSCs have also been reported to drive protective M2 macrophage polarization after brain trauma [109]. In mice with traumatic brain injuries and receiving intracerebroventricular human BM-MSCs, MSCs upregulated Ym1 and arginase-1 mRNA levels as well as the number of Ym1+ cells. Changes in M2 phenotype were associated with early and persistent recovery of neurological functions and reparative changes of the lesioned microenvironment.* In vitro*, MSCs increased Ym1 and CD206 mRNA levels and decreased expression of inducible nitric oxide synthase in primary murine macrophages. MSCs also reversed the proinflammatory response of primary murine macrophages induced by TNF-*α* + IL-17 or by TNF-*α* + IFN-*γ*.

## 11. Conclusion

MSCs can exert their immunomodulatory effect at multiple levels. MSCs regulate cells of both adaptive and innate immunity including T cells, B cells, DC, macrophages, and NK cells. MSCs provide macrophages with signals that stimulate the polarization of regulatory M2 phenotype. The elevated expression of M2 macrophages explains many of the beneficial effects observed with administration of the cells in animal models for wound healing, brain/spinal cord injuries, and diseases of heart, lung, and kidney. Further studies on the molecular mechanisms involved in the interaction between MSCs and macrophages will contribute to a better understanding of MSCs biology and the optimal use of MSCs in the clinical practice.
